# *pH* and *pCl* Operational Parameters in Some Metallic Ions Separation with Composite Chitosan/Sulfonated Polyether Ether Ketone/Polypropylene Hollow Fibers Membranes

**DOI:** 10.3390/membranes12090833

**Published:** 2022-08-26

**Authors:** Anca Maria Cimbru, Abbas Abdul Kadhim Klaif Rikabi, Ovidiu Oprea, Alexandra Raluca Grosu, Szidonia-Katalin Tanczos, Maria Claudia Simonescu, Dumitru Pașcu, Vlad-Alexandru Grosu, Florina Dumitru, Gheorghe Nechifor

**Affiliations:** 1 Analytical Chemistry and Environmental Engineering Department, University Politehnica of Bucharest, 011061 Bucharest, Romania; 2 Technical College of Al-Mussaib (TCM), Al-Furat Al-Awsat University, Babylon-Najaf Street, Najaf 54003, Iraq; 3 Department of Inorganic Chemistry, Physical Chemistry and Electrochemistry, University Politehnica of Bucharest, 011061 Bucharest, Romania; 4 Department of Bioengineering, University Sapientia of Miercurea-Ciuc, 500104 Miercurea-Ciuc, Romania; 5 Department of Electronic Technology and Reliability, Faculty of Electronics, Telecommunications and Information Technology, University Politehnica of Bucharest, 061071 Bucharest, Romania

**Keywords:** heavy metal ions separation, membrane permeation, composite membranes, sulfonated poly (ether ether ketone), polypropylene hollow fiber, chitosan, pH and pCl operational parameters

## Abstract

The development of new composite membranes is required to separate chemical species from aggressive environments without using corrective reagents. One such case is represented by the high hydrochloric acid mixture (very low pH and pCl) that contains mixed metal ions, or that of copper, cadmium, zinc and lead ions in a binary mixture (Cu–Zn and Cd–Pb) or quaternary mixture. This paper presents the obtaining of a composite membrane chitosan (Chi)–sulfonated poly (ether ether ketone) (sPEEK)–polypropylene hollow fiber (Chi/sPEEK/PPHF) and its use in the separation of binary or quaternary mixtures of copper, cadmium, zinc, and lead ions by nanofiltration and pertraction. The obtained membranes were morphologically and structurally characterized using scanning electron microscopy (SEM), high resolution SEM (HR–SEM), energy dispersive spectroscopy analysis (EDAX), Fourier Transform InfraRed (FTIR) spectroscopy, thermogravimetric analysis, and differential scanning calorimetry (TGA-DSC), but also used in preliminary separation tests. Using the ion solutions in hydrochloric acid 3 mol/L, the separation of copper and zinc or cadmium and lead ions from binary mixtures was performed. The pertraction results were superior to those obtained by nanofiltration, both in terms of extraction efficiency and because at pertraction, the separate cation was simultaneously concentrated by an order of magnitude. The mixture of the four cations was separated by nanofiltration (at 5 bars, using a membrane of a 1 m^2^ active area) by varying two operational parameters: pH and pCl. Cation retention could reach 95% when adequate values of operational parameters were selected. The paper makes some recommendations for the use of composite membranes, chitosan (Chi)–sulfonated poly (ether ether ketone) (sPEEK)–polypropylene hollow fiber (Chi/sPEEK/PPHF), so as to obtain the maximum possible retention of the target cation.

## 1. Introduction

Metals with atomic numbers (Z) greater than iron’s (Z = 26) are known as ”heavy metals”, and their technical and economic importance is so great that their per capita consumption was an indicator of quality of life during the period of intensive industrial development [1,2]. Of these metals, copper, cadmium, zinc, and lead have outstanding technical applications in electronics and electrical engineering, construction, transportation, the automotive industry, the dyes and pesticides industry, agriculture, and telecommunications [3,4,5]. The distribution map of these metals around the globe has changed so much that areas with natural reserves have less of these elements than the developed regions to which they were brought for use [6,7]. The chemical species deriving from these metals contaminate the environment so that they appear as polluting elements in water, soil, and even air [8,9]. It is not at all surprising that various wastes containing copper, cadmium, zinc, and lead are an important resource of raw materials, and the amount recycled from these metals has become a symbol and indicator of environmental protection [10]. The rich natural deposits were exploited by very well-developed techniques specific to metallurgy, but also to chemistry, electrochemistry, and hydrometallurgy (flotation, precipitation-sedimentation, extraction, ion exchange, electrolysis) [11,12,13]. Unfortunately, the recycling of industrial waste containing the considered metals is not completely solved due to the complexity of the various sources, as well as the different and very low concentrations [14,15]. The removal of heavy metal ions from various wastewaters, up to the limits accepted by international standards [16], uses conventional techniques, as well as the use of bio-adsorption [17,18], adsorption onto nanomaterials [19,20], ion–molecular flotation [21,22], magnetic separation [23,24], and numerous membranes and membrane processes [25,26,27,28,29,30].

Given the low concentration of metals considered in different wastes and wastewaters, their removal is often considered reasonable and an important goal/objective, the biggest challenge remaining their separation and recovery [31,32]. The requirements for recovery separation technologies are both process (selectivity and productivity) and technical–economic and environmental (reduction of material consumption and operating costs) [33,34]. 

The membrane technologies respond well to process requirements but are still being researched and developed to become cost-effective [35]. Among the many membrane materials used in the study of the separation and recovery of heavy metals, such as copper, cadmium, zinc, and lead, recent research has been focused on various nanoparticles [36,37,38], synthetic resins [39], and bio-sorbents [40]. Among polymeric membrane materials with ionic groups, sulfonated polyether-ether-ketone has more and more applications [41,42], and among bio-sorbents, cellulose and its derivatives, such as chitosan, are increasingly used [43,44,45].

In order to increase the process performance, the design of the membrane modules is of overwhelming importance and that is why, every time a specific separation is approached, research is carried out on flat, spiral, tubular, or hollow-fiber-type modules [46,47].

The amount of fluid and the concentration of metal ions in the feed phase can decide the type of module needed, but most of the time, for the upscaling of the processes, from lab to pilot scale and into production, the hollow-fiber modules are preferred [48,49].

The speciation of metals in the aqueous medium to be processed is another element that can determine the costs of a membrane process, both in terms of the required membrane materials and the configuration of the membranes in the permeation module [50].

The characteristics of wastewater or solutions obtained by acid leaching of industrial solid waste are dominated by two restrictive parameters, pH and pCl, and the intervention with reagents to regulate these parameters must be as economical as possible (low number of reagents and low concentrations) [51,52].

Starting from the performances of chitosan/sulfonated polyether ether ketone composite membranes in electromembrane processes, specifically in fuel cells, the present paper dealt with the preparation and characterization of a chitosan/sulfonated polyether ether ketone/polypropylene hollow-fiber (Chi/sPEEK/PPHF) composite membrane. For testing the composite membrane in separation processes such as pertraction and nanofiltration, acidic solutions containing copper, cadmium, zinc, and/or lead ions were considered. In the study, binary solutions containing pairs of cations (Cu^2+^-Zn^2+^ or Cd^2+^-Pb^2+^), but also aqueous solutions containing all four cations, were processed. 

This paper examined the recovery of copper, cadmium, zinc, and lead from synthetic solutions using composite membranes of chitosan/sulfonated polyether ether ketone/polypropylene hollow fiber (Chi/sPEEK/PPHF), using pH and pCl as operational parameters.

## 2. Materials and Methods

### 2.1. Reagents and Materials

#### 2.1.1. Reagents

CuSO_4_·5H_2_O, CdSO_4_, ZnSO_4_, Pb(NO_3_)_2_, Cu(NO_3_)_2_ ·3 H_2_O, Cd(NO_3_)_2_ ·4H_2_O, Zn(NO_3_)_2_, NaCl, chitosan, and glacial acetic acid (analytical grade, Sigma-Aldrich Chemie GmbH, Steinheim, Germany) were used in the studies. NaOH pellets, H_2_SO_4_ (96%), HCl 35% ultrapure and NH_4_OH 25% (analytical grade) were purchased from Merck KGaA Darmstadt, Germany. 

Ultrapure water was used for preparing the feed solutions. 

#### 2.1.2. Materials

The characteristics of the polymeric compounds and derivatives used in the study are presented in Table 1. 

The hollow polypropylene fibers used as support for membranes were provided by GOST Ltd., Perugia, Italy [56,57,58].

### 2.2. Procedures

#### 2.2.1. Sulfonated Polyether Ether Ketone (sPEEK) Preparation

Three hundred mL H_2_SO_4_ of 96% concentration was placed in a 500 mL glass vial with a sealing cap, after which 25 g of polymer (PEEK) was gradually added, stirring continuously, manually, to avoid agglomeration of the polymer. After about 2 h of stirring, the bottle with the polymer solution was kept without stirring for up to 24 h in order to promote complete dissolution of the polymer in acid. This time range should not be exceeded because additional sulfonation of the polymer would occur. After processing the reagent mass by removing traces of sediment and filtering suspended materials (gels or other impurities), a clear light orange solution of 4.4% sulfonated poly (ether ether ketone) (sPEEK) was obtained. The gravimetric percentage composition of the solution, determined by electrochemical titration, was 4.40% sulfonated poly (ether ether ketone) (sPEEK), 90.36% H_2_SO_4_, and 5.24% water.

**Figure 1:**
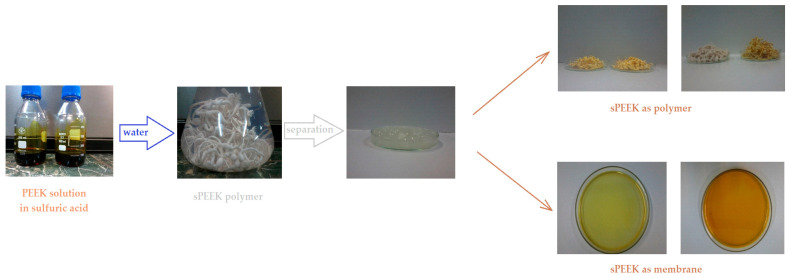
Scheme for obtaining sulfonated poly (ether ether ketone) sPEEK as a polymer and as a membrane from the solution of poly (ether ether ketone) (PEEK) in sulfuric acid.

According to the same method, a solution of 30 g PEEK and 300 mL of 96% sulfuric acid was prepared. A solution of 5.2% sPEEK concentration was obtained, with various degrees of sulfonation depending on the storage time of the solution. The gravimetric percentage composition of the solution, determined by electrochemical titration, was 5.20% sulfonated poly (ether ether ketone) (sPEEK), 89.33% H_2_SO_4_, and 5.47% water. The colors of the solutions varied from light orange to brown, indicating the different degree of sulfonation (Figure 1) [55,56].

The determination of the degree of sulfonation and the acidity index were reported [55,56,57], and their presentation can be easily followed electrochemically, but also chemically.

#### 2.2.2. Obtaining Composite Membranes

The solution of sulfonated poly (ether ether ketone) (sPEEK) was used as-is for impregnation of membranes used as such for impregnating hollow-fiber polypropylene membranes using the impregnation method [57,58].

sPEEK/HFPP (sulfonated poly (ether ether ketone)/polypropylene hollow-fiber) composite membranes can be conditioned either by drying or immersion in water or aqueous solutions. In order to obtain the chitosan/sulfonated poly (ether ether ketone)/polypropylene hollow-fiber Chi/sPEEK/PPHF composite membrane, the sulfonated poly (ether ether ketone)/polypropylene hollow-fiber sPEEK/PPHF composite membrane was immersed in a solution of 3% chitosan in 3% acetic acid [59]. Each type of membrane is washed with water and dried in vacuum for 24 h at 50 °C.

Drying of the obtained membranes is necessary for characterization, but for use in the separation tests this step can be avoided. However, the membrane polypropylene support is not affected by drying, being carried out at a moderate temperature and under vacuum. At the same time, the attack capacity of sulfuric acid is diminished by the presence of polyether ether ketone. 

Membrane materials are characterized by the determination of porosity [60], morphology [61], thermal characteristics [62], and the ion-exchange capacity and retention of heavy metal ions [63].

#### 2.2.3. Separation Techniques

The binary solution (Cu^2+^-Zn^2+^) can be prepared from the available reagents without restrictions, but the aqueous solutions containing Cd^2+^-Pb^2+^ of all four cations require precautions and therefore the corresponding nitrates were used.

The separation tests were performed with equimolar solutions of Cu(NO_3_)_2_, Cd(NO_3_)_2_, Zn(NO_3_)_2_, and Pb(NO_3_)_2_ obtained in ultrapure water [64].

**Figure 2:**
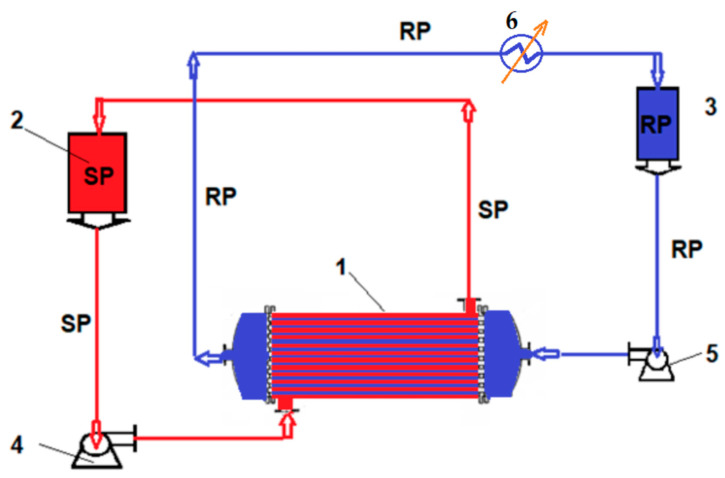
Schematic of the installation for pertraction: SP—source phase, RS—receiving phase. 1. Hollow-fiber pertraction module; 2. SP reservoirs; 3. RP reservoirs; 4. SP pump; 5. RP pump; 6. Thermostat.

**Figure 3:**
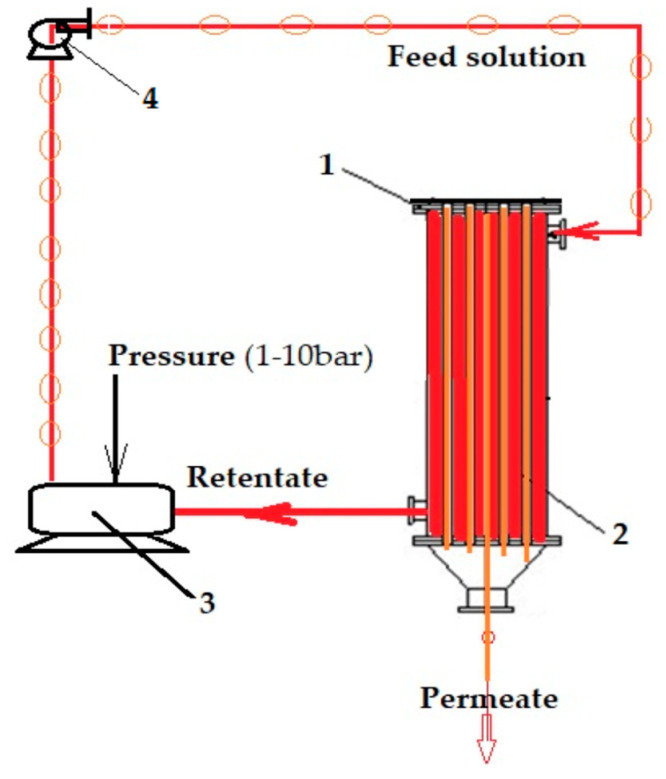
Schematic of the installation for nanofiltration: 1. Hollow fiber pertraction module; 2. Composite membranes; 3. Pressure reservoir; 4. Recycling pump.

In order to perform the tests, nanofiltration and pertraction experiments were performed in installations with a tubular configuration module [65], presented in Figure 2 and Figure 3. The volume of solution subjected to the experiments was 10 L, and the ion concentration was in the range of 10^−6^–10^−4^ mol/L. The chosen operational parameters were pH and pCl, the variation of which was conducted with hydrochloric acid, sodium hydroxide, or ammonia and sodium chloride. 

The operating conditions of pertraction are presented for each test performed separately, and the nanofiltration was carried out in the installation with the characteristics shown in Table 2. 

The pertraction installation has the same geometric characteristics of the membrane module (length, diameter, and membrane surface), but operates at atmospheric pressure. The circulation of the source phase was ensured by the volumetric recirculation pumping through the exterior of the hollow-fiber composite membranes, and of the receiving phase through its interior. The flow rate of the source phase was 100–1000 mL/min, and of the receiving phase was 10–100 mL/min.

### 2.3. Performances Materials, Membranes, and Processes Determination Procedure 

The fluxes from the source phase [55,56] were determined against the measured permeate mass within a determined time range, applying the following equation:

(1) $$J=\frac{M}{S\cdot t}\left(g/m^{2}h\right)$$

where: *M* = permeate mass (g), *S* = effective surface of the membrane (m^2^), *t* = the time necessary to collect the permeate volume (h).

The extraction efficiency (*EE*%) in the separation process or retention (*R*) in the nanofiltration process of analytes was calculated using the concentration or absorbance of the solutions [57,58]:

(2) $$EE\left(\%\right)\;or\;R\left(\%\right)=\frac{\left(c_{0}-c_{f}\right)}{c_{0}}\cdot 100$$

where *c_f_* is the final concentration of the solute (metallic ions), and *c*_0_ is the initial concentration of the solute (metallic ions).

The concentration of metal ions was determined spectrophotometrically for binary solutions, considering the additivity of the individual absorbances of the complexed ions (see Equation (3)). For the ternary system, the concentrations were determined through atomic absorption spectrometry (AAS) using the characteristic wavelengths:

(3) $$A_{t}=A_{1}+A_{2}$$

where *A_t_* is the overall absorbance, and *A*_1_ ad *A*_2_ are the specific absorbances of each ion at the chosen wavelength [62,64].

### 2.4. Equipment 

The scanning microscopy studies, SEM and HR-SEM were performed on a Hitachi S4500 system (Hitachi High–Technologies Europe GmbH, Mannheim, Germany) [66]. 

Thermal analysis (TG-DSC) was performed with a STA 449C Jupiter apparatus from Netzsch (NETZSCH-Gerätebau GmbH, Selb, Germany). Each sample weighed approximately 10 mg. The samples were placed in an open alumina crucible and heated up to 900 °C with 10 K∙min^−1^ rate, under flow of 50 mL∙min^−1^ dried air. As reference, we used an empty alumina crucible. The evolved gases were analyzed with a FTIR Tensor 27 from Bruker (Bruker Co., Ettlingen, Germany), equipped with a thermostat gas cell [67].

The UV–Vis analyses of the solutions were carried out on a CamSpec M550 Spectrophotometer (Spectronic CamSpec Ltd., Leeds, UK) [68]. 

The electrochemical processes were followed up with a PARSTAT 2273 Potentiostat (Princeton Applied Research, AMETEK Inc., Berwyn, PA, USA). A glass cell with a three-electrode setup was used [69].

The pH and pCl of the medium were followed up with a combined selective electrode (HI 4107, Hanna Instruments Ltd, Leighton Buzzard, UK) and a multi-parameter system (HI 5522, Hanna Instruments Ltd., Leighton Buzzard, UK) [70].

To assess and validate the content in metal ions, the atomic absorption spectrometer AAnalyst 400 AA Spectrometer (Perkin Elmer Inc., Shelton, CT, USA) with a single-element hollow-cathode lamp was used, driven by WinLab32–AA software (Perkin Elmer Inc., Shelton, CT, USA) [68,69,70].

## 3. Results and Discussions

Separation of low-concentration ions from various aqueous systems of complex composition is an important goal in the field of membranes and membrane processes. Heavy metal ion-poor systems have been treated with various membrane processes including reverse osmosis, direct osmosis, nanofiltration, dialysis, electrodialysis, or liquid membranes. Both the need to use a small amount and number of the reagents and recuperative separation continue to encourage research.

This paper used synthetic solutions that properly simulate aqueous solutions from the recovery of waste from electronics and electrical engineering, especially those from Cu–Zn and Pb–Cd batteries. Such solutions contain, for example, in concentrations of 10^−6^–10^−4^ mol/L, ions of copper, cadmium, zinc, and lead in the mixture.

The separation of the aqueous system was studied in hollow-fiber modules with composite membranes of sulfonated poly (ether ether ketone) (sPEEK)–polypropylene hollow fiber (sPEEK/PPHF) or chitosan (Chi)–sulfonated poly (ether ether ketone) (sPEEK)–polypropylene hollow fiber (Chi/sPEEK/PPHF).

The use of operational parameters pH and pCl is based on the equilibria (4)−(7) that were established in the hydrochloric supply solutions:

CuCl_2_ + 2 Cl^−^ ⇌ [CuCl_4_]^2−^_(aq)_ (4)

ZnCl_2_ + 2 Cl^−^ ⇌ [ZnCl_4_]^2−^_(aq)_ (5)

PbCl_2(s)_ + 2Cl^−^⇌ [PbCl_4_]^2−^_(aq)_ (6)

CdCl_2_ + 2Cl^−^⇌ [CdCl_4_]^2−^_(aq)_ (7)

These equilibria have been extensively studied [71,72,73] and chemical speciation plays a key role in the approached membrane separation processes.

### 3.1. Morphological, Structural and Thermal Characterization of the Obtained Composite Membranes

#### 3.1.1. Scanning Electron Microscopy Study

**Figure 4:**
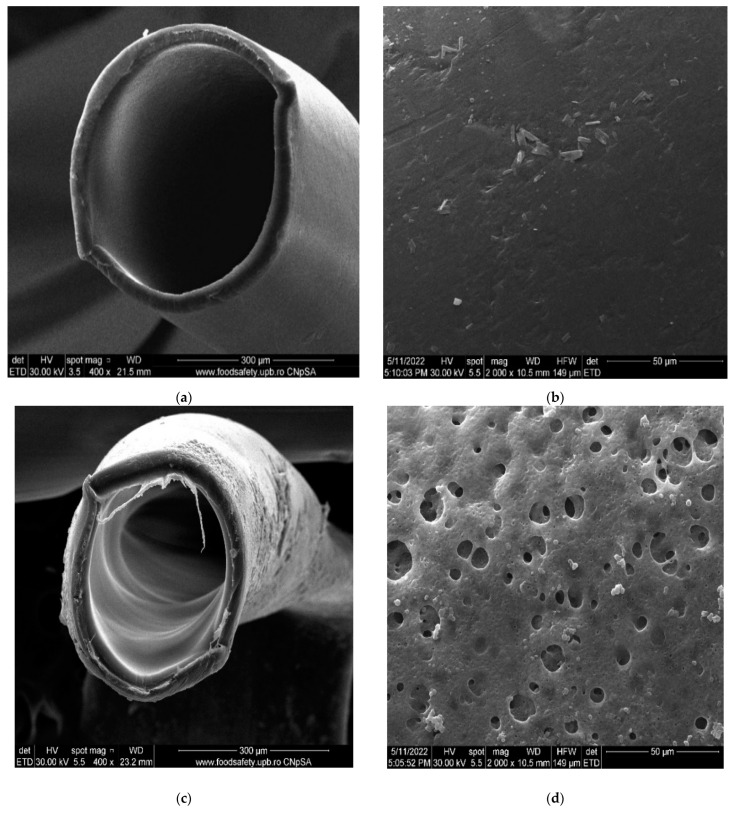

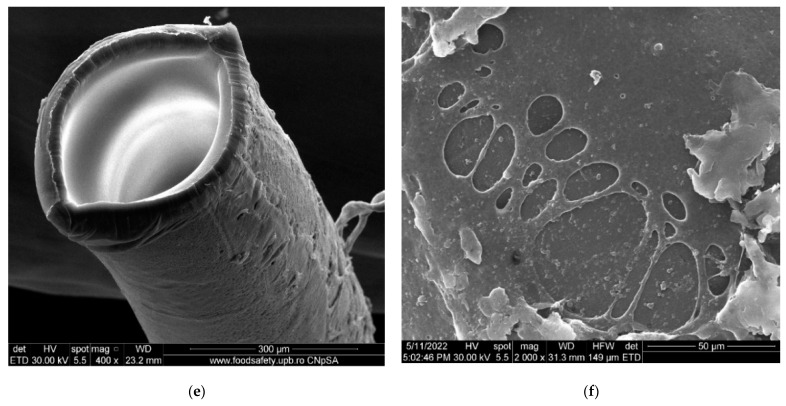
Scanning Electron Microscopy (SEM) on: (**a**) polypropylene hollow-fiber membrane (PPHF); (**b**) detail on surface; (**c**) sulfonated poly (ether ether ketone) (sPEEK)-polypropylene hollow fiber (sPEEK/PPHF); (**d**) detail on surface; (**e**) chitosan (Chi)–sulfonated poly (ether ether ketone) (sPEEK)–polypropylene hollow fiber (Chi/sPEEK/PPHF); (**f**) detail on surface.

**Figure 5:**
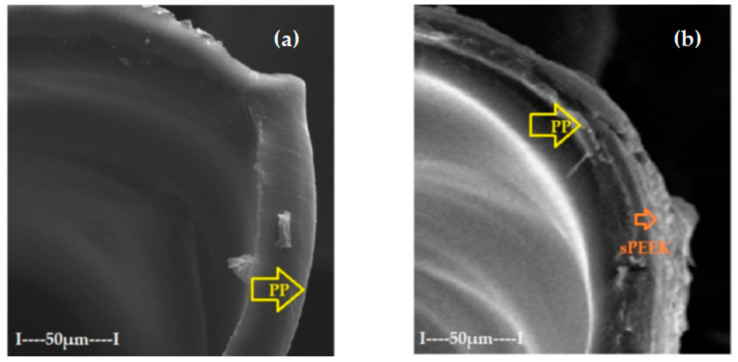
Scanning Electron Microscopy (SEM) details on polypropylene hollow-fiber membranes (PPHF) (**a**); and composite membranes (**b**).

The morphology obtained through scanning electron microscopy provides important information both for the operation for the conditioning of the membranes in the permeation module. Figure 3 shows sections and details of the composite membranes obtained. It can be observed that polypropylene hollow-fiber membrane (PPHF) (Figure 4a,b and Figure 5) had an inner diameter of about 300 µm, a thickness of the membrane wall of about 20 µm (Figure 4a and Figure 5), and a relatively smooth surface (Figure 3b). By depositing the layer of sulfonated poly (ether ether ketone) on the polypropylene support, a composite membrane (sSPEEK-PPHF) was obtained (Figure 4c,d and Figure 5) in which the layer of sulfonated poly (ether ether ketone) had a thickness of about 10 µm (Figure 4c and Figure 5), and the membrane surface had pores and micropores specific to ultrafiltration membranes [74]. Subsequent deposition of chitosan to obtain the composite membrane chitosan (Chi)–sulfonated poly (ether ether ketone) (sPEEK)–polypropylene hollow fiber (Chi/sPEEK/HFPP) did not significantly increase the thickness of the membrane wall (Figure 4e), but significantly changed the appearance of the surface composite fibers (Figure 4f).

When evaluating the layers of composite membrane the mode of examination by electron microscopy must be taken into account, under advanced vacuum. In such conditions, the only thickness that was maintained was that of the supporting polypropylene fiber (unaffected by the vacuum). In the normal state, the sPEEK or Chi/sPEEK layers of the composite membranes were strongly hydrated, while in vacuum they were completely dehydrated, thus affecting the illustrated results (see Figure 4c–f and Figure 5).

**Figure 6:**
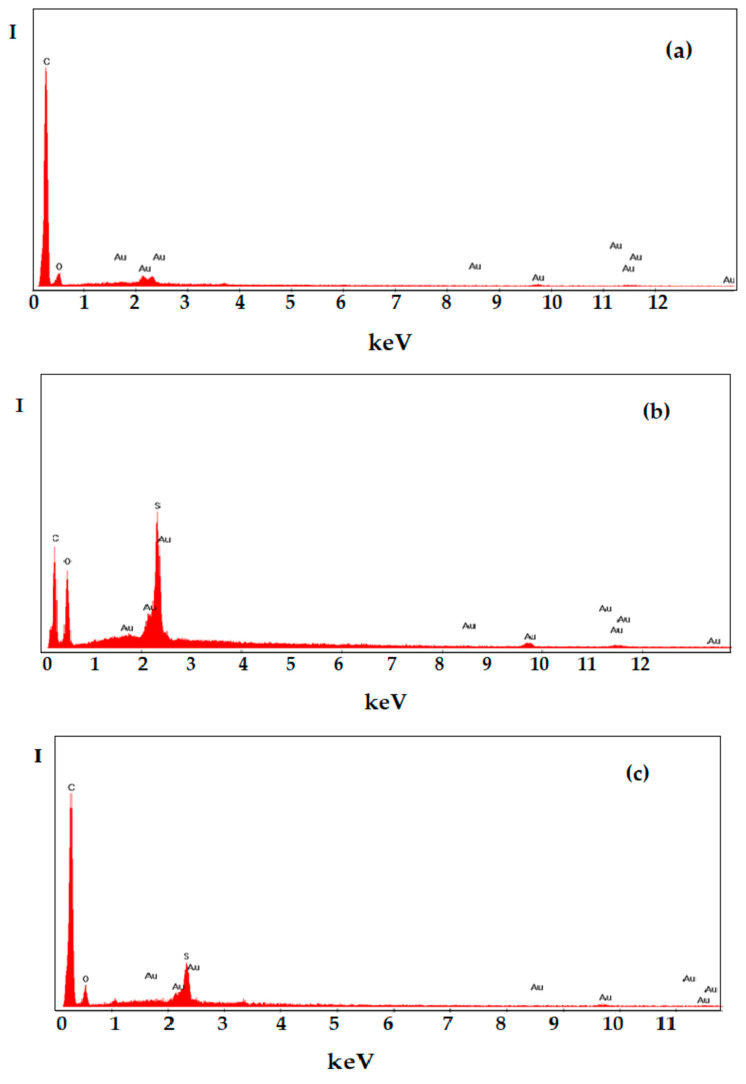
Energy dispersive X-ray analysis (EADX) on: (**a**) polypropylene hollow-fiber membrane (PPHF); (**b**) sulfonated polyether ether ketone (sPEEK)–polypropylene hollow fiber (sPEEK/PPHF); and (**c**) chitosan (Chi)–sulfonated poly (ether ether ketone) (sPEEK)–polypropylene hollow fiber (Chi/sPEEK/PPHF).

It is interesting that energy dispersive X-ray analysis (EADX) (Figure 6) highlights the change in surface composition. Thus, if only carbon and oxygen atoms appear in the polypropylene support (Figure 6a), in the composite membranes both the atomic ratio C:O and the composition change due to the appearance of sulfur atoms (Figure 6b,c). Although it is a strictly local analysis, energy dispersive X-ray analysis (EADX) is very useful to qualitatively follow the process of membrane formation as well as the process of separation of the complex system.

#### 3.1.2. Fourier Transform InfraRed (FTIR) and UV-Vis Spectrometry Analysis

**Figure 7:**
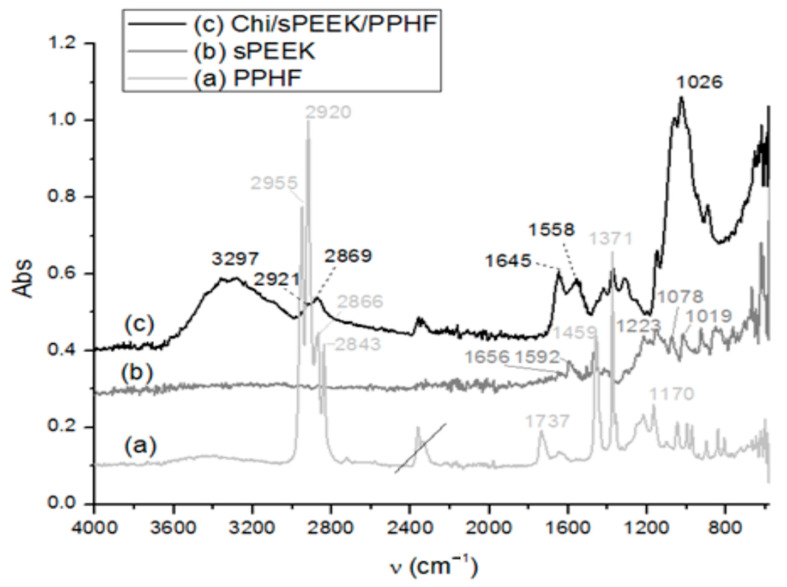
Fourier Transform InfraRed (FTIR) on (**a**) polypropylene hollow-fiber membrane (PPHF); (**b**) sulfonated poly (ether ether ketone) (sPEEK); and (**c**) chitosan (Chi)–sulfonated poly (ether ether ketone) (sPEEK)–polypropylene hollow fiber (Chi/sPEEK/PPHF).

**Figure 8:**
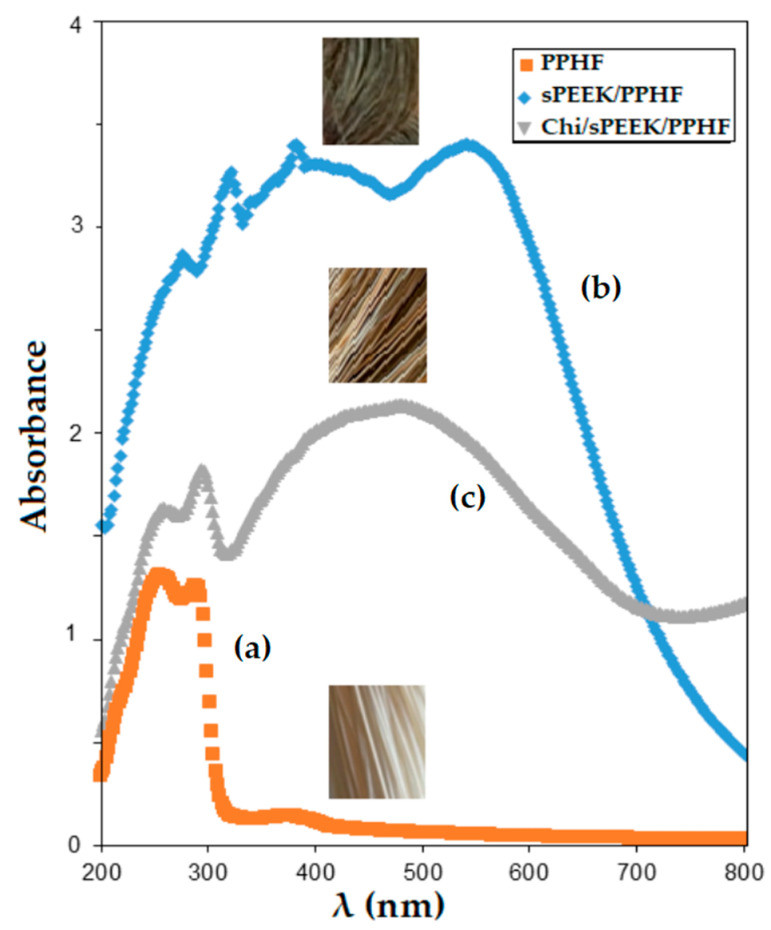
UV-Vis spectrometry on (**a**) polypropylene hollow-fiber membrane (PPHF); (**b**) sulfonated poly (ether ether ketone) (sPEEK)-polypropylene hollow fiber (sPEEK/PPHF); and (**c**) chitosan (Chi)–sulfonated poly (ether ether ketone) (sPEEK)–polypropylene hollow fiber (Chi/sPEEK/PPHF).

Structurally, the studied membranes have specific functions that could be highlighted by Fourier Transform InfraRed (FTIR) spectral analysis (Figure 7). At the same time, the UV-Vis spectrum indicated significant differences (Figure 8).

The FTIR spectra revealed, for each constituent of the composite membrane (Chi/sPEEK/PPHF), characteristic absorption bands; for sPEEK (Figure 7b)), one can observe the peak at 1656 cm^−1^ (as a small shoulder) assigned to the stretching mode of C=O, and the indicative peaks for the presence of O=S=O groups at 1223, 1078, and 1019 cm^−1^. The sharp peak at 1592 cm^−1^ (C=C) had a reduced intensity and this feature is attributed to the crosslinking of the sPEEK chains with oxygen, as described in the literature [75,76,77,78]. The PPHF FTIR-ATR spectrum (Figure 7a) showed peaks centered at 2955, 2920, 2866, and 2843 cm^−1^, very characteristic features of stretching vibration modes of -CH_2_-, -CH-, and -CH_3_ groups from polypropylene material. It is a known fact that the polypropylene polymer is susceptible to oxidation; therefore, in the PPHF spectrum one can observe a maximum at 1731 cm^−1^ corresponding to the formation of oxygen-containing groups. For the composite membrane (Chi/sPEEK/PPHF) (Figure 7c), a strong band centered at 3291 cm^−1^ corresponds to N-H and O-H stretching, as well as the intramolecular hydrogen bonds present in the chitosan structure. The absorption bands at around 2921 and 2869 cm^−1^ can be attributed to C-H symmetric and asymmetric stretching, respectively. The presence of residual N-acetyl groups was confirmed by the bands at around 1645 cm^−1^ (C=O stretching of amide I) and at 1558 cm^−1^ that correspond to the N-H bending of amide II. The strong band at 1026 cm^−1^ corresponds to C-O stretching and obscured the other characteristic bands of sPEEK and PPHF in this region. 

These characteristics are useful to the experimenter that will be able to effortlessly select the fibers needed to enter the specific permeation module. UV-Vis spectrometry examination may also be an indicator of membrane aging [78]. 

#### 3.1.3. Thermal Behaviour of Membrane Materials and Composite Membrane

Thermal analysis, TG-DSC (thermogravimetry and differential scanning calorimetry), was performed with a STA 449C F3 apparatus, from Netzsch (Selb, Germany), between 20 and 900 °C, in a dynamic (50 mL/min) air atmosphere. The evolved gases were analyzed with a FTIR Tensor 27 from Bruker (Bruker Co., Ettlingen, Germany) equipped with a thermostatic gas cell. 

**Figure 9:**
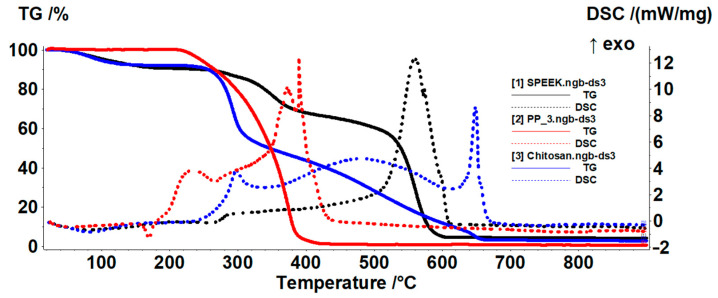
Comparative thermal diagrams of the three membrane materials.

The diagram of the three basic materials for obtaining composite membranes is presented as a whole in Figure 9.

**Figure 10:**
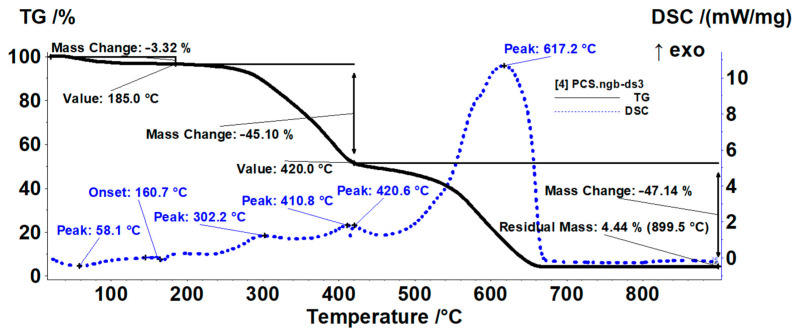
Detailed thermal diagrams of the composite membranes.

**Figure 11:**
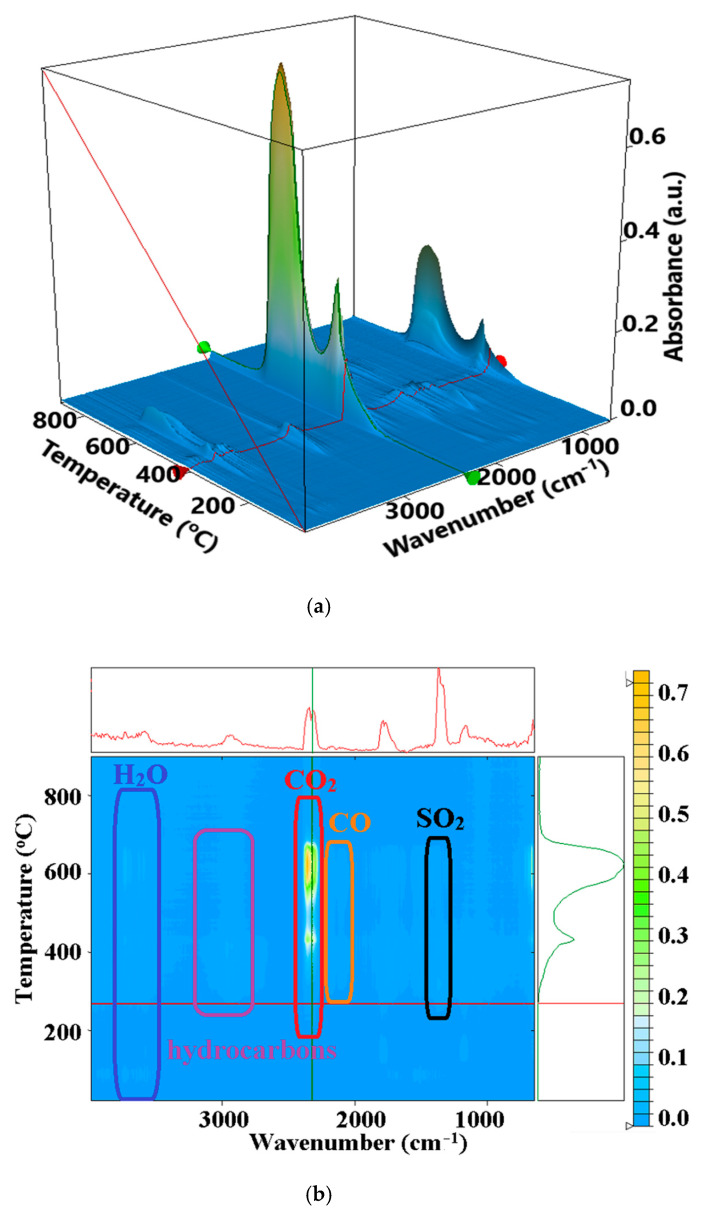
The evolved gases’ FTIR 3D diagram for the PCS sample (**a**); and its 2D projection with assigned identification/temperature intervals (**b**).

The composite membrane sample presented three mass loss steps on the TG curve (Figure 10). The first loss of 3.32% was recorded up to 185 °C. Two small endothermic effects accompanied the process. The first one, with a minimum at 58.1 °C, can be ascribed to the loss of some residual water molecules as the evolved gases FTIR analysis indicated (Figure 11). 

The melting of the PP fibers generated the second effect with the onset at 160.7 °C [68]. In the temperature interval 185–420 °C we recorded a mass loss of 45.10% in multiple, overlapped processes as indicated by the multiple peaks from DSC curve. Both PP and chitosan polymeric chains were broken in smaller fragments after 200 °C, and the oxidation of these fragments generated the overall exothermic small peak from 302.2 °C [79]. In parallel, sPEEK went through a desulfonation process. On the DSC curve the thermal effects overlapped, endothermic from decomposition and exothermic from oxidation, the resulting overall exothermic effect, indicating the prevalence of the oxidation processes. The exothermic peak from 410.8 °C is due to oxidation of the last PP fragments [70]. The last mass loss step, representing 47.14%, took place after 420 °C when slow oxidation of chitosan and sPEEK fragments was completed, coupled with the burning of the carbonaceous residual mass from 617.2 °C.

The 3D FTIR plot (Figure 11a) presents the evolution of the FTIR spectrum vs. temperature. By projecting this map in 2D space (wavenumber vs. temperature), we can easily identify the components and temperature intervals when they are eliminated from the sample (Figure 11b). The FTIR spectra recorded for the evolved gases indicate the presence of water at low temperature (under 100 °C) and some traces of acetic acid over 150 °C. In the second mass-loss step, over 185 °C the FTIR analysis of evolved gases indicated the presence of CO_2_, H_2_O, and traces of CO, which are normal components for the oxidation reaction of organics. We also identified the presence of SO_2_ from sPEEK desulfonation (the absorption band from 1381 cm^−1^), acetic acid from chitosan decomposition (the absorption bands from 1796 and 1180 cm^−1^), and other saturated and unsaturated hydrocarbons (the absorption bands around 3000 cm^−1^).

### 3.2. Preliminary Ion Separation Tests with Prepared Composite Membranes

The recuperative separation of copper, cadmium, zinc, and lead ions was studied by two membrane processes: nanofiltration and pertraction (Figure 2 and Figure 3), using binary systems: copper–zinc and lead–cadmium, as well as quaternary systems: copper, cadmium, zinc, and lead.

The nanofiltration or pertraction module had a usable membrane area of 1 m^2^, the difference in behavior of the membranes being imposed by the forced circulation under pressure (nanofiltration) or flow through the outside of the membrane of the source phase with the capture of the ions transported in the receiving phase inside the membrane (pertraction) (Figure 2 and Figure 3). 

The operation in the nanofiltration process was performed under a pressure of 4−6 bar, with a recirculation flow rate of 0.1–1 L/min for the source phase through the exterior of the composite hollow-fiber membrane (Table 2, Figure 3). 

In the case of pertraction, the module played the role of the contactor. The source phase circulated through the outside of the composite hollow-fiber membrane at a flow rate of 0.1–1 L/min, and inside them the receiving phase had a flow rate of 0.10–0.100 L/min (Figure 2).

The membranes used in the separation modules were chitosan (Chi)–sulfonated poly (ether ether ketone) (sPEEK)–polypropylene hollow fiber (Chi/sPEEK/PPHF), but comparative tests were also performed with sulfonated poly (ether ether ketone) (sPEEK)–polypropylene hollow fiber (sPEEK/PPHF), at the same time or alternately.

The variable operational parameters were pH and pCl, trying to use as few reagents as possible, considering the supply with a strong acidic solution that also contains excess chloride ions (coming from both hydrochloric acid and sodium chloride). 

The pH was adjusted either with sodium hydroxide solution or with ammonia, which is also formed in elution systems.

#### 3.2.1. Nanofiltration Separation of Binary Systems with Composite Membranes from 3 mol/L Hydrochloric Acid Solutions

The first system studied consisted of a 3 mol/L hydrochloric acid solution containing copper and zinc ions in an equimolar mixture of 10^−4^ mol/L concentration. This system can be separated with chitosan membranes (Chi)–sulfonated poly (ether ether ketone) (sPEEK)–polypropylene hollow fiber (Chi/sPEEK/PPHF), since sulfonated poly (ether ether ketone) (sPEEK)–polypropylene hollow fibers (sPEEK/PPHF) are unable to interact with [MCl_4_]^2−^ anions.

Copper and zinc form with hydrochloric acid complex combinations of different stabilities depending on their concentration equilibria (4) and (5). Because in the presence of 3 M HCl zinc forms a complex [ZnCl_4_]^2−^ anion, while copper remains in the form of Cu^2+^ cations or less stable complexes, the two ions can be separated by passage through a module with a membrane of chitosan (Chi)–sulfonated poly (ether ether ketone) (sPEEK)–polypropylene hollow fiber (Chi/sPEEK/PPHF). The complex anion [ZnCl_4_]^2−^ will be retained on the membrane while Cu^2+^ will pass through the module without being retained, the tetra-chloro cupper complex being much less stable [80]. 

**Figure 12:**
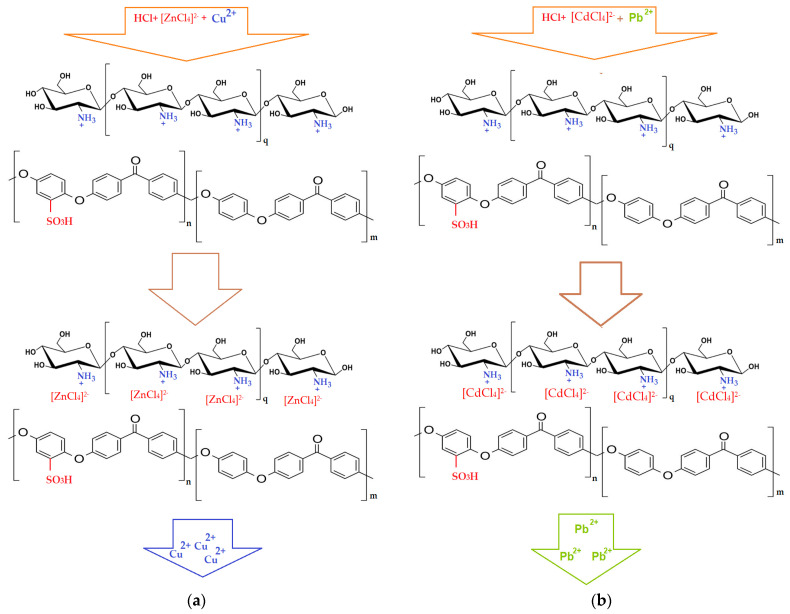
Separation of the mixture of copper and zinc ions or cadmium and lead with a strong acid solution (3 mol/L hydrochloric acid) by nanofiltration copper and zinc ions (**a**); and cadmium and lead ions (**b**).

Elution of Zn^2+^ from the module was conducted in the presence of HCl 3⋅10^−2^ M, which no longer provided conditions for the existence of complex anions and Zn^2+^ cations left the membrane (Figure 12). The scheme shown in Figure 12a is specific to the nanofiltration of chitosan (Chi)–sulfonated poly (ether ether ketone) (sPEEK)–polypropylene hollow-fiber (Chi/sPEEK/PPHF) membranes.

The second system studied consisted of a 3 mol/L hydrochloric acid solution containing lead and cadmium ions in an equimolar mixture of 10^−4^ mol/L concentration.

The lead chloride was insoluble and that of cadmium was very weakly dissociated, but in strongly hydrochloric solution (3 mol/L HCl) and at a temperature of 50 °C, by nanofiltration through chitosan (Chi)–sulfonated poly (ether ether ketone) (sPEEK)–polypropylene hollow-fiber (Chi/sPEEK/PPHF) membranes, the cadmium as an anion [CdCl_4_]^2−^ was retained while the lead passed into the permeate as lead cations (Figure 12b).

**Figure 13:**
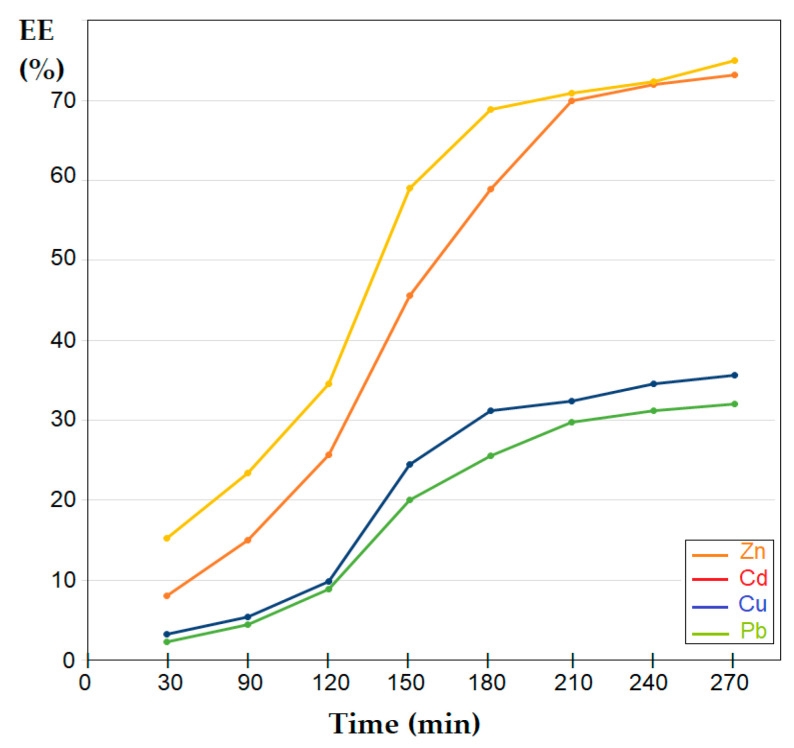
Retention–elution curves when separating the ion mixture with a strongly acidic solution (3 mol/L hydrochloric acid) by nanofiltration (5 bars and 200 mL/min feed solution flow) for the copper–zinc system and the lead–cadmium system from an equimolar 10^−4^ mol/L solution.

The appearance of the retention–elution curves in the case of nanofiltration (5 bars and 200 mL/min feed solution flow) indicated a worse performance when separating lead and copper ions compared with that of cadmium and zinc ions (Figure 13). The recuperative separation of zinc and cadmium ions, which form more stable tetra-chloro complexes, after 4 h of operation, exceeded 70%, while for copper and especially lead ions it did not reach 40%. One explanation for these results would be that the membrane, however, retains some of the lead or copper ions either in the form of anion complexes or expels them (does not allow passage through the membrane) as positive ions.

The results in a single separation stage are promising, especially since the concentration of test ions in the feed was relatively high, i.e., 10^−4^ mol/L.

#### 3.2.2. Pertraction Separation of Binary Systems with Composite Membranes from 3 mol/L Hydrochloric Acid Solutions

**Figure 14:**
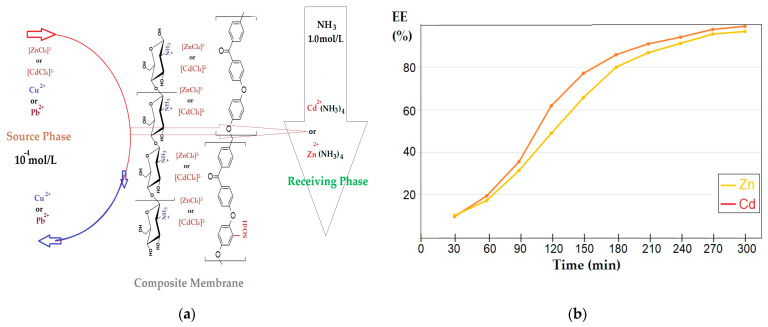
Separation of the mixture of equimolar concentration of 10^−4^ mol/L of the copper and zinc ions, or lead and cadmium ions, from the strongly acidic source phase (3 mol/L hydrochloric acid) by pertraction in the ammonia-receiving phase (NH_3_ aqueous solution 1.0 mol/L): (**a**) trans-membrane transport scheme; (**b**) the efficiency of cadmium or zinc ion extraction.

Figure 14a shows the separation of the copper–zinc system of equimolar concentration 10^−4^ mol/L through membranes of chitosan (Chi)–sulfonated poly (ether ether ketone) (sPEEK)–polypropylene hollow fiber (Chi/sPEEK/PPHF) in the pertraction module in which 1.0 mol/L ammonia receptor solution flowed through the fibers, which contributes to the fixation of zinc ions as a tetra-ammonia ion. The process control was performed by monitoring the concentration of zinc ions in the receiving phase, the end of the process being considered when reaching the degree of recovery of 90% of zinc. Unlike nanofiltration, in this process, the tetra-chloro-zincate ion passed through the membrane, and the cupric ion remained mostly in the supply.

On one hand, this behavior is justified by the particular mechanism of pertraction, in which the cationic groups of chitosan in the strongly acidic environment favor the transport of the more stable anion, ZnCl_4_^2−^, but also the fact that copper ions, Cu^2+^, reaching the interface with the basic ammonia solution are retained by the membrane which, in that section, will have the free amino groups.

The lead–cadmium system behaved in the same way (Figure 14a), thus confirming the proposed transport mechanism (Figure 14b). After four hours of operation, the recovery of the two ions in the receiving solution exceeded 90%. It is noteworthy that the cadmium ion separated with noticeably better efficiency from its lead system than the zinc ion from the zinc–copper system.

The advantage of the extraction process, from the point of view of the higher extraction efficiency, is doubled by the fact that the receiving solution, being 10 times smaller in volume than the receiving solution (1L NH_3_ aqueous solution 1.0 mol/L), led to the concentration of the separate chemical species. An important disadvantage of extraction is the additional consumption of reagents (ammonia).

#### 3.2.3. Separation of Quaternary Systems with Composite Membranes by Nanofiltration

If the source of the aqueous solution from which the copper, zinc, cadmium, and lead ions are to be recovered has a pH that falls within the normal pH scale, as is the case for surface waters contaminated with the ions considered, the use of nanofiltration through the prepared composite membranes can have as operational parameters both the pH of the feed and the pCl (salinity induced with sodium chloride). 

Table 3 presents the main parameters of copper, zinc, cadmium, and lead ions in aqueous solutions [80,81] of variable pH and pCl considered in establishing the operating parameters for the nanofiltration of solutions containing a mixture of these ions. 

**Figure 15:**
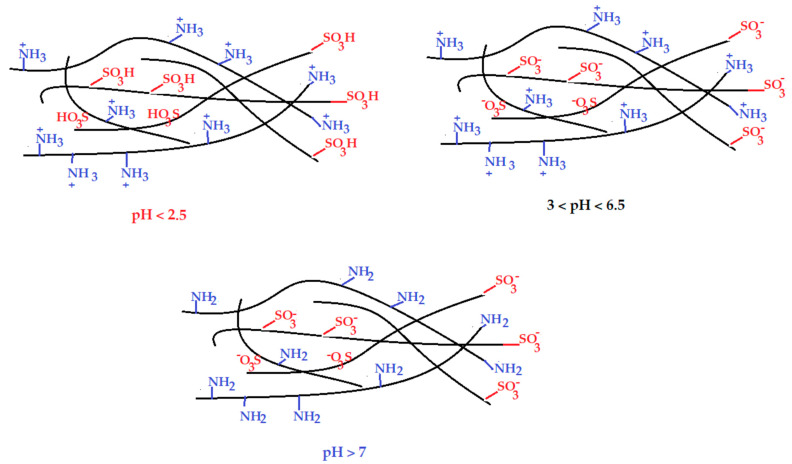
Scheme of the ionic charge of the functional groups of the composite membrane depending on the pH.

These parameters must be correlated with the ionic charge of the functional groups of the composite membrane (Table 2), depending on the pH of the aqueous environment in which it operates, as follows: at pH up to 1.9, the functional groups were in the form –SO_3_H and –NH^3+^; between 2 and 6.4 were–SO^3−^ and –NH^3+^; and after pH 6.5 the groups became –SO^3−^ and NH_2_ (Figure 15).

At the same time, the way in which the pH variation is achieved can influence the efficiency of the separation (retention) of the four cations in the nanofiltration process of 10 L equimolar solution 10^−4^ mol/L ions of copper, zinc, cadmium, and lead, at 5 bars with chitosan (Chi)–sulfonated poly (ether ether ketone) (sPEEK)–polypropylene hollow-fiber (Chi/sPEEK/PPHF) composite membranes with active surfaces of 1 m^2^, at a recirculation rate of 0.20 L/min.

The pH variation can be achieved by neutralizing the initial stock solution of zero pH (1 mol/L HCl solution) with solid sodium hydroxide or using only hydrochloric acid. In the first case, the pH varied from 0 to 8, and pCl remained identical, and in the second case both pH and pCl had identical values (Table 4).

The obtained results showed that the ion retention depended on both pH and pCl, because in the aqueous solution there were competitive equilibria of formation of chlorides (MCl_2_), tetra-chloro-complexes ([MCl_4_]^2−^), hydroxides (M(OH)_2_), or even aqua complexes and/or hydroxy complexes. The data in Table 2 are a good benchmark for justifying the retention values, but they are not enough because the interaction of each species with the membrane is complex and very different from case to case. This is also due to the fact that the tasks of the functional groups vary with the change of the pH of the supply solution.

Some recommendations regarding the separation of copper, zinc, cadmium, and lead ions from a mixture with membranes of chitosan (Chi)–sulfonated poly (ether ether ketone) (sPEEK)–polypropylene hollow fiber (Chi/sPEEK/PPHF) can be as follows:

- The separation of zinc and cadmium ions showed the narrowest variation range, most likely because these ions were retained to the same extent either by the cationic or anionic groups of the composite membrane.
- Copper separation was excellent at pCl and pH values as high as possible.
- The separation of lead in environments with sufficiently low pCl was little-influenced by the pH value. However, caution is advised when both pH and pCl are high.
- At high pH values, the separation of copper, zinc, and cadmium ions was very good because they interacted with the membrane in both sulfonic and amino groups.

**Figure 16:**
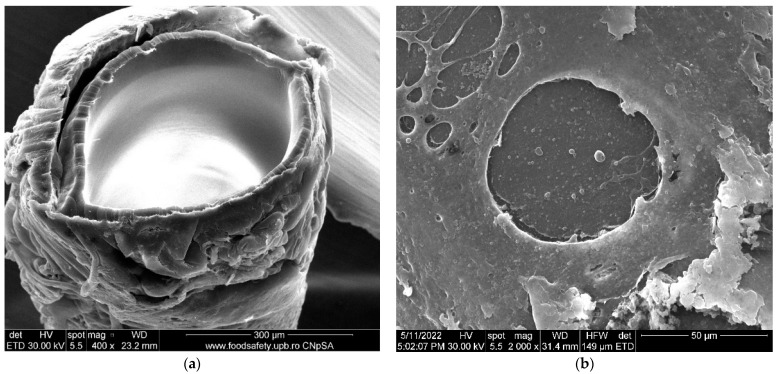
Scanning electron microscopy (SEM) for repeatedly used membranes: (**a**) section; and (**b**) surface.

It should be noted that in repeated uses the membranes lose their qualities (retention decreases for all cations studied), most likely due to the detachment of the composite membrane from the support (Figure 16).

The application of nanofiltration for the separation of the quaternary system must take into account the permeate flows of the composite membrane chitosan (Chi)–sulfonated poly (ether ether ketone) (sPEEK)–polypropylene hollow fiber (Chi/sPEEK/PPHF), which were much lower than the polypropylene support (PPHF) or the sulfonated poly (ether ether ketone) (sPEEK)–polypropylene hollow-fiber (sPEEK/PPHF) membrane (Table 5).

The support polypropylene membrane is a membrane specific to microfiltration and has relatively low water flows, being hydrophobic. Although the sulfonated poly (ether ether ketone) (sPEEK)–polypropylene hollow-fiber (sPEEK/PPHF) membrane had the pores of the support covered, by increasing the pressure, specific ultrafiltration flows were obtained. However, it must be borne in mind that at the contact surface with the supply it had the hydrophilized layer of sulfonated polyether ether ketone (sPEEK). Finally, the composite membrane of chitosan (Chi)–sulfonated poly (ether ether ketone) (sPEEK)–polypropylene hollow fiber (Chi/sPEEK/PPHF) had specific nanofiltration flows, and the operation at 5−6 bar was performed only for technical and economic reasons (obtaining an acceptable flow at medium working pressure).

At this stage of the study, the increase in pressure above 6 bar was not taken into account both for energy consumption reasons but also because the composite membrane must be optimized by a possible crosslinking to avoid detachment from the support (Figure 16). 

Among the objectives to be achieved by developing the study of this type of membrane, the following must be found:

- Tracking the influence of the molecular weight of chitosan;
- Degree of sulfonation of the poly-ether-ether-ketone;
- Decreasing the thickness of the polymer membrane support layer;
- Optimization of flow-retention under the imposed pressure limit conditions.

## 4. Conclusions

In this study, we presented the obtaining of a composite membrane of chitosan (Chi)- sulfonated poly (ether ether ketone) (sPEEK)–polypropylene hollow fiber (Chi/sPEEK/PPHF), which was characterized morphologically, structurally, and from the point of view of the separation performances of copper, cadmium, zinc, and lead ions, in either binary mixture (Cu–Zn and Cd–Pb) or quaternary mixtures, in the conditions of strongly hydrochloric systems (very low pH and pCl). The separation of binary systems from solutions of hydrochloric acid 3 mol/L was performed both by nanofiltration and pertraction. The quaternary system was separated by nanofiltration under variable pH and pCl conditions.

The obtained membranes were morphologically and structurally characterized by scanning electron microscopy (SEM), high-resolution SEM (HR–SEM), energy dispersive spectroscopy analysis (EDAX), Fourier Transform InfraRed (FTIR) spectroscopy, thermal gravimetric analysis, and differential scanning calorimetry (TGA).

Preliminary separation tests showed that binary systems could be efficiently separated by both nanofiltration and pertraction. Extraction would be more advantageous in terms of separation efficiency (90% is reached), but also because the chemical species that is extracted is concentrated by almost an order of magnitude.

Nanofiltration has the advantage of a simpler operation and applicability to multiple systems, but the separation efficiency is strongly influenced by both pH and pCl.

Depending on the target cation and the pH and pCl conditions, retentions of over 90% (for Pb) and almost 95% (for Cu) could be obtained.

The study carried out here opens the perspective of obtaining and characterizing new chitosan (Chi)–sulfonated poly (ether ether ketone) (sPEEK)–polypropylene hollow-fiber (Chi/sPEEK/PPHF) composite membranes using different types of chitosan, but also sulfonated polymer compounds that improve not only the stability and lifetime of the membranes but also of the process performances.

## Figures and Tables

**Table 1:** The characteristics of the used polymeric and derivative compounds.

Polymer Compounds	Symbol	Molar Mass (Da)	Solubility	pKa *
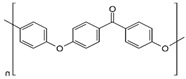	PEEK	30.000	Sulfuric acid	-
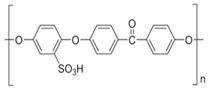	sPEEK		Organic polar solvents	1.9
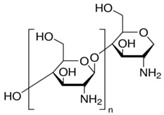	Chi		Acidulated water	6.5

* pK_a_ values as reported in scientific literature [53,54,55]

**Table 2:** The characteristics of the nanofiltration installation used.

Module Type	Module Length (cm)	Module Diameter (cm)	Operating Pressure (bar)	Membrane Length (cm)	Membrane Surface (m^2^)	Feed Solution Flow (mL/min)
Hollowfiber membrane	75 ± 1.0	6 ± 0.1	1–10	75 ± 1.0	1.0 ± 0.1	100–1000

**Table 3:** The characteristics of the tested metallic ions in aquatic solution.

Metallic Ion	Ionic Radius (Å)	[MCl4]^2−^ pK _instability_	Precipitation Hydroxide pH	MCl_2_ Solubility in Water (g/100 mL)	Ks M(OH)_2_
Cu^2+^	1.96	5.30	4.4	75.7	1·10^−20^
Zn^2+^	0.83	0.15	6.8	432.0	5·10^−17^
Cd^2+^	0.94	2.46	4.5	119.6	1·10^−14^
Pb^2+^	1.81	13.22	4.2	0.99	3·10^−16^

**Table 4:** Separation efficiency (retention) of copper, zinc, cadmium, and lead ions from aqueous solutions, depending on the pH and pCl.

Feed Solution Characteristics		Metallic Ion Retention R (%)			
pH	pCl	Cu^2+^	Zn^2+^	Cd^2+^	Pb^2+^
0	0	23.12	75.33	74.67	87.58
1	0	29.34	62.42	70.84	89.23
4	0	48.90	45.20	54.08	92.00
6	0	79.85	40.48	59.65	90.23
8	0	93.32	64.84	61.20	70.05
1	1	18.56	60.32	65.44	85.00
4	4	68.26	49.86	51.62	60.88
6	6	90.45	62.95	74.32	50.08
8	8	95.18	78.59	80.51	48.82

**Table 5:** Transmembrane water flows for the studied membranes.

Pressure (bar)	Flux of Pure Water (L/m^2^·h)		
	PPHF	sPEEK/PPHF	Chi/sPEEK/PPHF
1.0	8.20	-	-
1.5	12.37	-	-
2.0	19.90	-	-
2.5	22.85	-	-
3.0	-	2.32	-
3.5	-	3.48	0.63
4.0	-	5.95	1.45
5.0	-	6.64	2.32
6.0	-	7.22	2.51

## Data Availability

Data are contained within the article.
